# Validation of the Linearity in Image Reconstruction Methods for Speckle Contrast Optical Tomography

**DOI:** 10.1109/jstqe.2025.3581407

**Published:** 2025-06-19

**Authors:** Alexander C. Howard, Byungchan Kim, Laura Carlton, Meryem A. Yücel, Bingxue Liu, David A. Boas, Xiaojun Cheng

**Affiliations:** The authors are with the Department of Biomedical Engineering, Neurophotonics Center, Boston University, Boston, MA 02215 USA

**Keywords:** Brain imaging, speckle-based techniques, diffuse optics, modeling, image reconstruction

## Abstract

Speckle contrast optical spectroscopy (SCOS) is an optical technique capable of measuring human cerebral blood flow and brain function non-invasively. Its tomographic extension, speckle contrast optical tomography (SCOT), can provide blood flow variation maps with measurements using overlapping source-detector channel pairs. Linearity is often assumed in most image reconstruction methods, but non-linearity could exist in the relations between measured signals and blood flow variations. We have constructed a forward model for SCOT using the Rytov approximation to solve the correlation diffusion equation and compared it with the first Born approximation as well as the more accurate, but computationally expensive Monte Carlo simulation approach. We have shown that the results obtained using the Rytov approximation are in good agreement with the Monte Carlo simulations, while the first Born approximation deviates from the other two methods for large blood flow variations. For instance, the first Born approximation breaks down at around 30% cerebral blood flow (CBF) changes within a volume of size 60 × 50 × 40 mm^3^, therefore we recommend using the Rytov approximation above this threshold. We have shown that our defined blood flow index (BFi) measured in SCOT is linearly related to local CBF variations, thus the forward and inverse problems can be solved linearly using the sensitivity matrix approach. We have then demonstrated image reconstruction experimentally showing human brain activations using our recently developed high-density SCOS system. Our method guides experimental system design and data analysis for SCOT.

## I. Introduction

Speckle contrast optical spectroscopy (SCOS) is an emerging camera-based optical technique that measures human cerebral blood flow (CBF) and brain function non-invasively [1], [2], [3], [4], [5], [6], [7], [8], [9], [10], [11], [12], [13]. In SCOS, the spatial speckle contrast K=std(I)/⟨I⟩ is calculated, where std(I) is the standard deviation and ⟨I⟩ is the mean of the speckle intensity. The reduction of K within a particular camera exposure time $T_{\text{exp}}$ can be utilized to quantify CBF. Recently, SCOS has been demonstrated to achieve an order of magnitude higher signal-to-noise ratio with a lower cost than traditional diffuse correlation spectroscopy (DCS) which is the current state-of-the-art optical technique to measure human CBF [6], [14]. Its tomographic extension, speckle contrast optical tomography (SCOT), has also been developed which measures the map of CBF changes with overlapping source-detector channel pairs [15], [16], [17]. In the pioneering work of SCOT [15], Varma et al. have developed the forward model using the first Born approximation, assuming the fractional change of the speckle contrast $\left(rK^{2}-1\right)$ is linearly related to the fractional CBF changes $\left(rD_{b}-1\right)$, where $D_{b}$ is the Brownian diffusion coefficient of the red blood cells (RBCs). Here r denotes the relative change of a variable, for instance, $rK^{2}=K^{2}/K_{0}^{2}$, where $K_{0}^{2}$ is the baseline value of $K^{2}$. For linear systems, a sensitivity matrix is constructed relating $\left(rK^{2}-1\right)$ measured in each source-detector pair and $\left(rD_{b}-1\right)$ in each voxel inside the tissue, and image reconstruction is obtained by solving the inverse problem. Other works using diffuse correlation tomography (DCT) [18] and SCOT [19] to measure blood flow in bone have employed the Rytov approximation. However, the assumption of linearity has not been validated under various conditions relevant to human brain function measurements for these methods.

In this manuscript, we built on the pioneering work of SCOT [15] by constructing a forward model to solve the correlation diffusion equation (CDE) using the Rytov approximation, taking into account higher-order dynamic scattering contributions, and compared it with the first Born approximation and Monte Carlo simulations. We see that for small CBF changes, either in a small localized region or a small $\left(rD_{b}-1\right)$, the results obtained using the first Born, Rytov, and Monte Carlo methods are in good agreement. But, for larger CBF changes, the first Born approximation deviates from Rytov and Monte Carlo methods. We found that $\left[r\left(1/K^{2}\right)-1\right]$ exhibits better linearity than $\left(rK^{2}-1\right)$ with $rD_{b}-1$, the latter of which is typically used in SCOT, while the former of which is traditionally used for estimating CBF changes in laser speckle contrast imaging (LSCI) [20]. We have also validated spatial linearity by exploring the signals arising from CBF changes in two local inclusions in the tissue, as well as the linearity in the reconstructed versus the true $rD_{b}$ with a source-detector array. We then demonstrated image reconstruction of the map of $rD_{b}-1$ during the brain activation in experiments using the linear method with the sensitivity matrix obtained using the Rytov approximation. We made the code for the forward model publicly available [21].

## II. Methods

### A. Correlation Perturbation Theory

We start with the theory of CDE. The unnormalized temporal field auto-correlation function is

(1) $$G_{1}(\tau )=\left\langle E^{*}(t)E(t+\tau )\right\rangle$$

Here E(t) is the electric field at time t. In a diffusive dynamic scattering medium, $G_{1}(\tau )$ follows the CDE measured at location r in the form of the Helmholtz equation [22], [23],

(2) $$\left[\nabla^{2}+C^{2}\left(\tau \right)\right]G_{1}\left(r_{s},r,\tau \right)=-\frac{vS}{D_{p}}\delta^{3}\left(r-r_{s}\right),$$

where $C^{2}(\tau )=v\left[\mu_{a}+\frac{1}{3}\mu_{s}^{\prime }k_{0}^{2}\left\langle \Delta r^{2}(\tau )\right\rangle \right]/D_{p}$, $r_{s}$ is the location of the source, S is the strength of the light source. Here v is the speed of the light in the medium, $\mu_{a}$ the absorption coefficient, $\mu_{s}^{\prime }$ the reduced scattering coefficient, $k_{0}=\frac{2\pi }{\lambda }$ the wavevector, λ the wavelength, $D_{p}=v/\left(3\mu_{s}^{\prime }\right)$ the photon diffusion coefficient, and $\left\langle \Delta r^{2}(\tau )\right\rangle$ the mean square displacement of scatterers, e.g. moving RBCs. Here $\left\langle \Delta r^{2}(\tau )\right\rangle =6D_{b}\tau$. In this manuscript, we ignore the convective motion of RBCs since it has been demonstrated that in human brain measurements, shear-induced diffusion dominates in the decay of the field auto-correlation function [24], [25], [26]. The analytical solution for a homogeneous semi-infinite medium has the well-known expression

(3) $$G_{1}\left(r_{s},r_{d},\tau \right)=\frac{3S{\mu^{\prime }}_{s}}{4\pi }\left[\frac{\text{exp}\left(-Cr_{1}\right)}{r_{1}}-\frac{\text{exp}\left(-Cr_{2}\right)}{r_{2}}\right].$$

Here $r_{1}={\left(\rho^{2}+z_{0}^{2}\right)}^{1/2}$, $r_{2}={\left(\rho^{2}+{\left(z_{0}+2z_{b}\right)}^{2}\right)}^{1/2}$, $z_{0}=1/\mu_{s}^{\prime },z_{b}=(5/3)\mu_{s}^{\prime }$, $\rho =\left|r_{s}-r_{d}\right|$ is the source-detector separation. We use the first Born and Rytov approximation to solve the equation with a perturbation $\Delta D_{b}\left(r^{\prime }\right)=D_{b}\left(r^{\prime }\right)-D_{b,0}\left(r^{\prime }\right)=\left(rD_{b}-1\right)D_{b,0}$ at a location $r^{\prime }$ inside the medium, where $D_{b,0}\left(r^{\prime }\right)$ is the baseline value of $D_{b}\left(r^{\prime }\right)$.

In the first Born approximation, the solution to (2) after the perturbation is expressed as the sum of the baseline $G_{1}^{0}\left(r_{s},r_{d},\tau \right)$ and a perturbation term

(4) $$G_{1}\left(r_{s},r_{d},\tau \right)=G_{1}^{0}\left(r_{s},r_{d},\tau \right)\left[1+\Phi_{s}\left(r_{s},r_{d},\tau \right)\right],$$

Here $\Phi_{s}\left(r_{s},r_{d},\tau \right)$ can be obtained from the Green’s function method by convolving the Greens’ function with the perturbed term

(5) $$\Phi_{s}\left(r_{s},r_{d},\tau \right)=-\frac{\int d^{3}r^{\prime }\frac{2v{\mu^{\prime }}_{s}k_{0}^{2}\tau \Delta D_{b}\left(r^{\prime }\right)}{D_{p}}H\left(r^{\prime },r_{d},\tau \right)G_{1}^{0}\left(r_{s},r^{\prime },\tau \right)}{G_{1}^{0}\left(r_{s},r_{d},\tau \right)}.$$

where $H\left(r^{\prime },r_{d},\tau \right)$ is the Green’s function for (2) and $H\left(r^{\prime },r_{d},\tau \right)=\frac{D_{p}}{vS}G_{1}^{0}\left(r^{\prime },r_{d},\tau \right)$. Compared to the first Born approximation that only considers first-order weak scattering, the Rytov approximation takes into account the higher-order scattering between the measured signal and $\Delta D_{b}$ as

(6) $$G_{1}\left(r_{s},r_{d},\tau \right)=G_{1}^{0}\left(r_{s},r_{d},\tau \right)exp\left[\Phi_{s}\left(r_{s},r_{d},\tau \right)\right],$$

with $\Phi_{s}\left(r_{s},r_{d},\tau \right)$ calculated in (5). We see that the first Born approximation in (4) is the first order Taylor expansion of the Rytov approximation in (6), i.e. $G_{1}^{0}\left(r_{s},r_{d},\tau \right)exp\left[\Phi_{s}\left(r_{s},r_{d},\tau \right)\right]=G_{1}^{0}\left(r_{s},r_{d},\tau \right)\left(1+\Phi_{s}\left(r_{s},r_{d},\tau \right)+\cdots \right)$. The normalized field autocorrelation function is obtained as

(7) $$g_{1}\left(r_{s},r_{d},\tau \right)=G_{1}\left(r_{s},r_{d},\tau \right)/G_{1}\left(r_{s},r_{d},0\right).$$

For SCOS/SCOT measurements, we obtain the speckle contrast K=std(I)/⟨I⟩ measured at a camera exposure time $T_{\text{exp}}$ as

(8) $$K^{2}\left(r_{s},r_{d},T_{exp}\right)=\frac{2\beta }{T_{exp}}\int_{0}^{T_{exp}}{\left|g_{1}\left(r_{s},r_{d},\tau \right)\right|}^{2}\left(1-\frac{\tau }{T_{exp}}\right)d\tau ,$$

If $g_{1}(\tau )$ follows the analytical expression of $g_{1}(\tau )=exp\left(-\tau /\tau_{c}\right)$, which is true for most human brain measurements [27], where $\tau_{c}$ is the decay constant, the contrast is expressed in terms of $\tau_{c}$ and $T_{\text{exp}}$ as

(9) $$K^{2}\left(T_{exp}\right)=\beta \frac{\tau_{c}}{T_{exp}}\left[1+\frac{\tau_{c}}{2T_{exp}}\left(\text{exp}\left(\frac{-2T_{exp}}{\tau_{c}}\right)-1\right)\right].$$

In this manuscript, we will conduct numerical integration using (8) to calculate $K^{2}$. But from (9), we see that $K^{2}\sim \tau_{c}\sim 1/D_{b}$ when $T_{\text{exp}}\gg \tau_{c}$. This suggests that $\left(r\left(1/K^{2}\right)-1\right)$ could exhibit better linearity with $\left(rD_{b}-1\right)$ than $rK^{2}-1$ utilized in the prior studies of SCOT [15].

### B. Monte Carlo Simulation

We compare the results with our previously developed Monte Carlo simulations [25], [28], [29]. To summarize, photons are launched at a source position, and a detector is placed at a distance away from the source on the surface of the sample, collecting re-emitted photons. For the $n^{\text{th}}$ photon arriving at the detector, we record its total pathlength $L_{n}$ and the accumulated dimensionless momentum transfer $Y_{n}$. The momentum transfer is the change of the wavevector during a single scattering event $\vec{q}={\vec{k}}_{\text{out}}-{\vec{k}}_{\text{in}}$, where ${\vec{k}}_{\text{in}}$ and ${\vec{k}}_{\text{out}}$ are the incident and scattered wavevectors respectively. Only elastic scattering is considered here, thus $\left|{\vec{k}}_{\text{in}}\right|=\left|{\vec{k}}_{\text{out}}\right|=k_{0}$. The accumulated dimensionless momentum transfer of a detected photon is the normalized sum of the momentum transfer of all the scattering events $Y_{n}=\sum q^{2}/2k_{0}^{2}$. With $Y_{n}$ and $L_{n}$ obtained with Monte Carlo simulations, the temporal field autocorrelation function can be calculated as

(10) $$g_{1}\left(\tau \right)=\frac{C}{N_{p}}\overset{N_{p}}{\underset{n=1}{\sum }}\text{exp}\left(-\frac{1}{3}Y_{n}k_{0}^{2}\langle \Delta r^{2}\left(\tau \right)\rangle \right)\text{exp}\left(-\mu_{a}L_{n}\right),$$

where C is a normalization factor such that $g_{1}(0)=1,N_{p}$ is the total number of the photons detected. For activation that happens in a localized region, the change of $\left.\Delta \left(Y_{n}\Delta r^{2}(\tau )\right\rangle \right)=Y_{n}\left(\Delta D_{b}\tau \right)$ can be obtained within the activation volume and applied to (10) for the photons that have been dynamically scattered within the activation region. More details can be found in [27]. Therefore, Monte Carlo simulations compute $g_{1}(\tau )$ from scattering events of every photon, which serves as the ground truth to test the analytical results obtained with the first Born (4) and Rytov approximations (6).

Another Monte Carlo simulation is used to approximate the autocorrelation from each source to each voxel in an anatomical head model [30]. This gives $H\left(r^{\prime },r_{d},\tau \right)$, $G_{1}^{0}\left(r_{s},r^{\prime },\tau \right)$, and $G_{1}^{0}\left(r_{s},r_{d},\tau \right)$ enabling the calculation of the perturbation term in (5). Due to the larger volume of the head model, we follow the methods described by Lin et al. [31]. In brief, the absorption term in an existing head model is replaced by the effective absorption term in (2),

(11) $$\mu_{a,eff}=\mu_{a}+2\alpha \mu_{s}^{\prime }k_{0}^{2}D_{b}\tau .$$

The fluence at location $r^{\prime }$ obtained at different τ values for a given source location $r_{s}$ gives $G_{1}\left(r_{s},r^{\prime },\tau \right)$. The simulation is then iterated over 129 values of τ, starting at 0s and followed by a logarithmically spaced range from 10^−7^ s to 4 × 10^−3^ s, where 4 × 10^−3^ s is the exposure time used in our experimental SCOS measurements. Since multiple τ values require multiple instances of the simulations, it is more time-consuming compared to the method used in (10); however, it enables simulation of the autocorrelations in the full volume without saving the momentum transfer of each photon in every voxel space, which is more efficient when constructing the sensitivity matrix. We used $k_{0}=7375\;\text{rad}/\text{mm}$ that corresponds to a wavelength of 852nm and the refractive index n=1.33 for the whole simulation volume. To obtain the sensitivity matrix for the experimental results, we utilized a real head geometry, instead of the homogeneous semi-infinite medium used to compare with analytical solutions, to better mimic experimental conditions. The optical properties of the head model [30] which serve as inputs to the Monte Carlo simulation are listed in Table I. We used the $\alpha D_{b}$ values reported by Lin et al. for the dynamic properties of the head [31].

### C. Image Reconstruction Theory

The sensitivity matrix A relating $r\left(1/K^{2}\right)-1$ and $rD_{b}-1$ can be defined as

(12) $$r\left(1/K^{2}\left(r_{s},r_{d};r^{\prime }\right)\right)-1=A\left(r_{s},r_{d};r^{\prime }\right)\left(rD_{b}\left(r^{\prime }\right)-1\right).$$

**Fig. 1.**
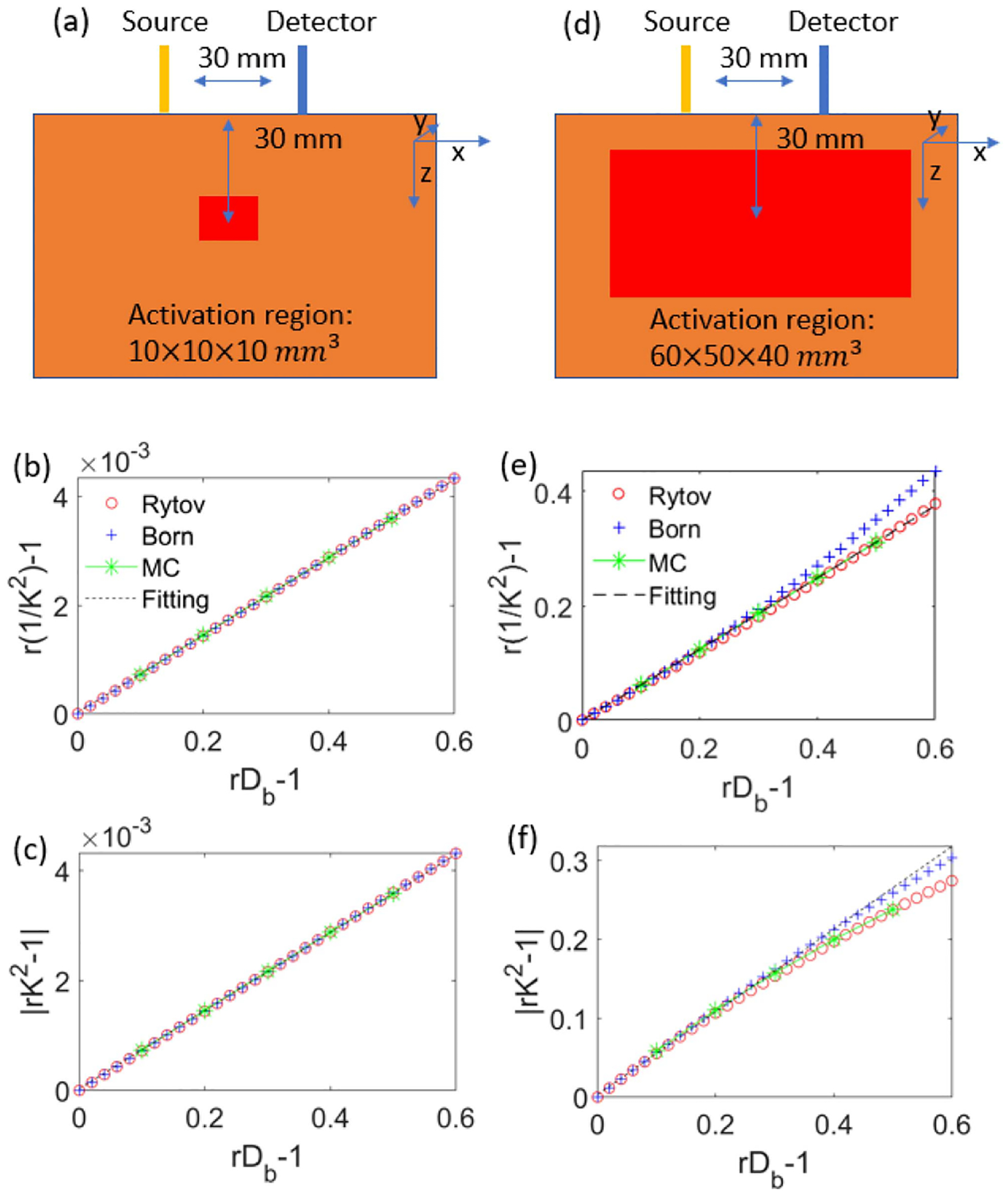
Validation of the linearity in SCOS signal versus blood flow changes. The comparison of the results obtained from Monte Carlo simulations (MC), the first Born approximation, and Rytov approximation are shown. Linear fitting of the MC results is also shown to demonstrate any deviations from linearity from these methods. (a)–(c) The (a) schematic plot and fractional change of the SCOS signal (b) $r\left(1/K^{2}\right)-1$ and (c) $rK^{2}-1$ versus the fractional CBF change, $rD_{b}-1$, within a small inclusion in the tissue (spanning $10\times 10\times 10\;mm^{3}$ centered at depth z=30mm and x=15mm from the source location). (d)–(f) The (d) schematic plot and fractional change of the SCOS signal (e) $r\left(1/K^{2}\right)-1$ and (f) $rK^{2}-1$ versus the fractional CBF change, $rD_{b}-1$, within a large inclusion in the tissue (spanning 60 × 50 × 40 mm^3^ centered at depth z=30mm and x=15mm from the source location). The source-detector separation is 30 mm. Other parameters used in the simulations $\mu_{s}^{\prime }=1\;mm^{-1}$, $\mu_{a}=0$, $D_{b,0}=10^{-6}{mm}^{2}/s$, wavelength λ=800nm, $T_{\text{exp}}=2\;\text{ms}$, β=1, voxel size for simulations $1\;{mm}^{3}$. In the Monte Carlo simulations, 10^8^ photons are used as collimated beam incidence.

**Fig. 2.**
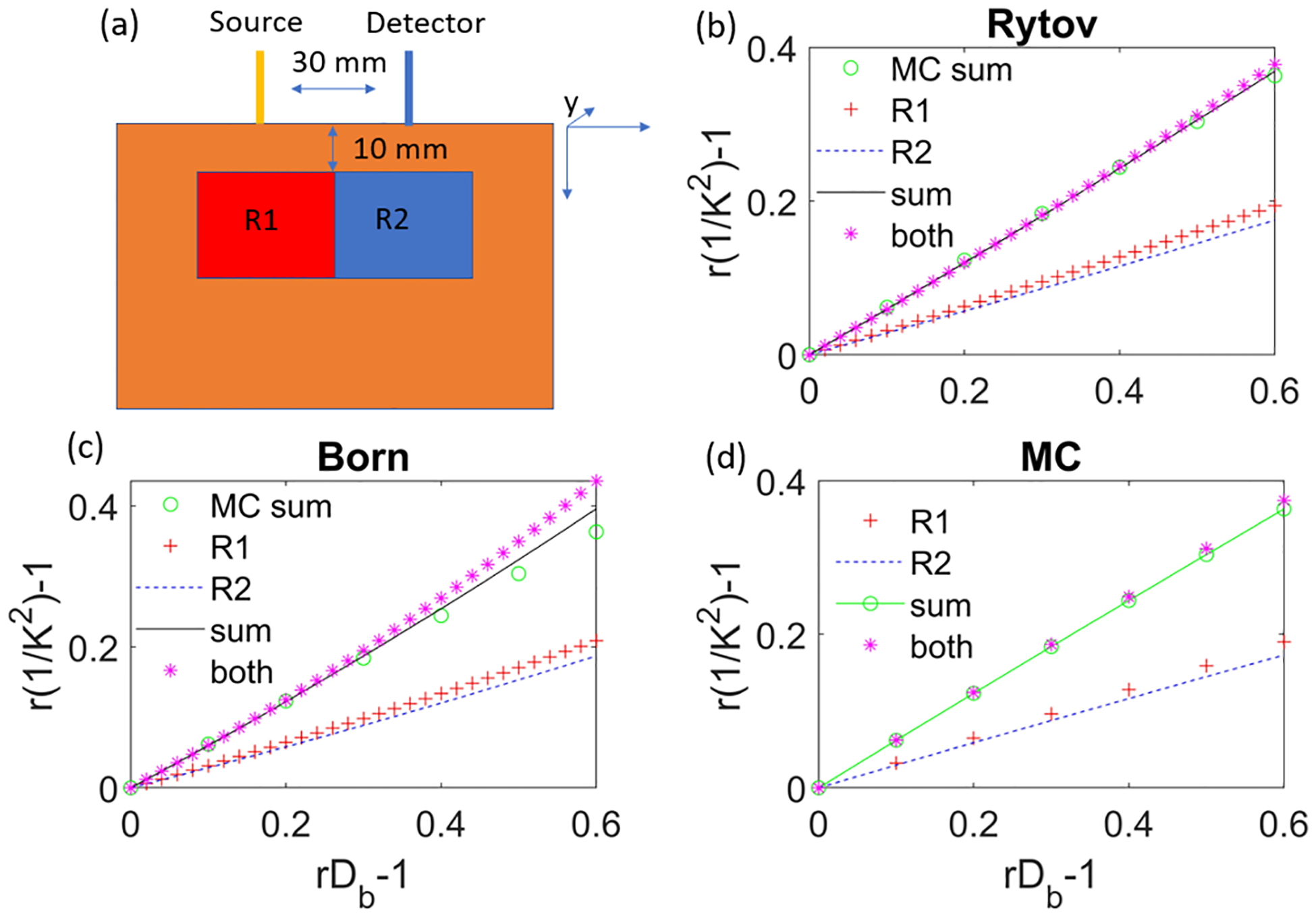
Validation of spatial linearity in SCOT. (a) Illustration of the detection system. The source-detector separation is 30 mm, and two adjacent regions (R1, R2) within the medium are activated, i.e $D_{b}$ changes within the region, individually and simultaneously. Both regions are of size $30\times 50\times 40{mm}^{3}$, and the top of these regions are 10mm away from the surface of the sample. (b)-(d) Results for $r\left(1/K^{2}\right)-1$ versus $rD_{b}-1$ with brain activation in R1 (red +), R2 (blue dashed line), the sum of the signals from R1 and R2 respectively (black solid line), and the signal arising from activation in both regions simultaneously (magenta *), obtained from (b) Rytov approximations, (c) the first Born approximation, and (d) Monte Carlo simulations. The sum of the signals for Monte Carlo simulations (Green) is also shown in (b) and (c) for comparison. Other parameters are the same as those used in Fig. 1.

Here $r\left(1/K^{2}\left(r_{s},r_{d};r^{\prime }\right)\right)$ is the relative change of $1/K^{2}\left(r_{s},r_{d}\right)$ when there is a localized blood flow change $rD_{b}\left(r^{\prime }\right)$ at the voxel at $r^{\prime }$ for a fixed exposure time $T_{\text{exp}}$. We use the subscript i to denote the ith source-detector pair $\left(r_{s},r_{d}\right)$, and j to denote the jth voxel inside the medium at a particular position $r^{\prime }$. Each element (i,j) in the matrix $A_{i,j}$ is expressed as $A_{i,j}=\left(r\left(1/K_{i,j}^{2}\right)-1\right)/\left(rD_{b,j}-1\right)$. Thus, we see that $A_{i,j}$ can be obtained from the slope of the $r\left(1/K^{2}\right)-1$ versus $\left(rD_{b}-1\right)$ as shown in Figs. 1–2 for a particular source-detector separation and activation region. For the experimental data, we have calculated a perturbation at every voxel $r^{\prime }$,

(13) $$\Phi_{s}\left(r^{\prime },r_{s},r_{d},\tau \right)=-\frac{\Delta V\left(r^{\prime }\right)r^{\prime }\frac{2v{\mu^{\prime }}_{s}k_{0}^{2}\tau \Delta D_{b}\left(r^{\prime }\right)}{D_{p}}H\left(r^{\prime },r_{d},\tau \right)G_{1}^{0}\left(r_{s},r^{\prime },\tau \right)}{G_{1}^{0}\left(r_{s},r_{d},\tau \right)},$$

where $\Delta V\left(r^{\prime }\right)$ is the voxel size. The perturbation term is projected onto a brain surface mesh [30] reducing the number of total elements in $A_{i,j}$. We then calculate $r\left(1/K^{2}\left(r_{s},r_{d};r^{\prime }\right)\right)-1$ using (6)–(8), (14).

Note that A is also proportional to the voxel size utilized in the integration in (5). Experimentally, $r\left(1/K^{2}\right)-1$ is measured and the spatial activation map $rD_{b}-1$ can be obtained by solving the inverse problem similar to what has been done in diffuse optical tomography (DOT) [32], [33], [34]

(14) $$rD_{b}-1={\left(A^{T}A+\lambda I\right)}^{-1}A^{T}\left(r\left(1/K^{2}\right)-1\right)$$

Here $A^{T}$ is the transpose of the matrix A, $rD_{b}$ and $r\left(1/K^{2}\right)$ are vectors with dimensions of the number of voxels and the number of source-detector pairs respectively, λ a scalar regularization parameter and I the identity matrix. λ is given by $\lambda =\alpha \text{max}\left[\text{diag}\left(A^{T}A\right)\right]$, where $\text{max}\left[\text{diag}\left(A^{T}A\right)\right]$ is the maximum of the diagonal elements of $A^{T}A$ and α is a normalized scalar regularization parameter [32], and α is set to be 0.001 for simulation results. For experimental results, we used a higher value of α=0.005 due to increased measurement noise.

### D. Experimental Methods

For experimental data collection, we used our recently developed multi-channel SCOS system [6], consisting of 17 cameras and 7 sources, to conduct human brain function measurements. An 852 nm VHG laser is temporally multiplexed to 7 source fibers using a galvanometer [35]. The optodes are aligned on the head using the same NinjaCap used for fNIRS measurement [36], and positioned by aligning anatomical markers with EEG 10–10 electrode positions marked on the cap [37]. Spatial statistics are calculated from the speckle images acquired by the cameras, and subtraction of shot noise, read noise, quantization noise, and noise from spatial heterogeneity is conducted as describe in our previous publications [6], [35].

### E. Word Color Stroop Task

Functional data is obtained from measurements during a word color stroop (WCS) task, adapted from Jahani et al. [38]. Briefly, the task consisted of two conditions. During the congruent (easy) condition, two words describing a color are shown, and the subject is asked to select which word’s text color matches its meaning. During the incongruent (hard) condition, one word is shown at the bottom, and three words are shown above. The subject is asked to select the word in the top row describing the color of the text below. The experimental procedure and protocols were approved and carried out in accordance with the regulations of Institutional Review Board of Boston University. Each participant provided a signed written informed consent form prior to the experiment.

## III. Results

### A. Numerical Simulation

We first show in Fig. 1 the comparison of the solutions obtained using the Rytov approximation, the first Born approximation, and Monte Carlo simulations for the brain activation geometries shown in Fig. 1(a), (d). We see that for a blood flow change within a small inclusion (Fig. 1(a)–(c)), the results obtained from these three methods are in good agreement, and both $r\left(1/K^{2}\right)-1$ and $rK^{2}-1$ are linearly related to $\left(rD_{b}-1\right)$. This is expected since for a function y(x), a small variation Δx induces a signal change Δy can be approximated by the first-order Taylor series. However, for a large inclusion, as shown in Fig. 1(d)–(e), we see that the Rytov result more accurately agrees with the Monte Carlo simulations and $r\left(1/K^{2}\right)-1$ is linearly related to $rD_{b}-1$ for both of these methods. The first Born approximation deviates from the Monte Carlo and Rytov approximation since it only considers small changes of $\Phi_{s}$ in (5). We calculated the percent error at different $rD_{b}$ values as $\frac{\left[r\left(1/K^{2}\right)-1\right]-\left[r\left(1/K_{\text{linear}}^{2}\right)-1\right]}{\left[r\left(1/K_{\text{linear}}^{2}\right)-1\right]}\times 100\%$, where $r\left(1/K_{\text{linear}}^{2}\right)-1$ is the linear fit of the Monte Carlo results, and $r\left(1/K^{2}\right)-1$ is the result obtained using MC, Rytov and Born approximations respectively. We consider a deviation greater than 5% to be significant. For a large inclusion (Fig. 1(e)), the first Born approximation $r\left(1/K^{2}\right)-1$ result deviates from the linear fit by greater than 5% for $rD_{b}-1>0.32$, while the results from both Monte Carlo and Rytov remain within 5% of the linear fit for all tested values of $rD_{b}-1$. This deviation is physiologically relevant, as the prior SCOS literature has reported an $rD_{b}-1$ close to 1 during the systolic peak of cardiac pulsation [6]. Comparing Fig. 1(e) and (f), we also see that $r\left(1/K^{2}\right)-1$ exhibits better linearity than $rK^{2}-1$, as expected from (9). Thus, it is desirable to use $r\left(1/K^{2}\right)-1$ instead of $rK^{2}-1$ to construct a linear forward model relating the SCOT signal and blood flow changes $\left(rD_{b}-1\right)$.

Another type of linearity that is often overlooked is the spatial linearity, i.e, the sum of the signal changes arising from brain activation in Region 1 and Region 2 (denoted as R1 and R2) respectively should be the same as the total signal change arising from activation in both regions simultaneously. Taking the first Born approximation as an example, we see that the approximation is valid for a small inclusion, Fig. 1(b), but not valid for a large inclusion, Fig. 1(e), which indicates that the spatial linearity breaks down for the first Born approximation when adding the responses from smaller regions. The validation of spatial linearity is demonstrated in Fig. 2. Here we used two relatively large volumes of activation regions $\left(30\times 50\times 40\;{mm}^{3}\right)$, since for small activation regions the results behave linearly (evidenced in Fig. 1(a)–(c)). We compare the results obtained from the sum of $r\left(1/K^{2}\right)-1$ arising from brain activation-induced blood flow changes $rD_{b}-1$ in R1 and R2 separately denoted as $\left(r\left(1/K_{\text{sum}}^{2}\right)-1\right)$, and $r\left(1/K^{2}\right)-1$ arising from activation in both R1 and R2 simultaneously denoted as $\left(r\left(1/K_{\text{both}}^{2}\right)-1\right)$. We see that results obtained from Rytov and Monte Carlo simulations exhibit good spatial linearity when comparing the sum of the signal changes from the two regions (black dashed line) and the total signal change when both regions are activated (magenta *), while the spatial linearity for the first Born approximation is compromised at large $rD_{b}-1$ values. The percent error is calculated as $\frac{\left[r\left(1/K_{\text{sum}}^{2}\right)-1\right]-\left[r\left(1/K_{\text{both}}^{2}\right)-1\right]}{\left[r\left(1/K_{\text{both}}^{2}\right)-1\right]}\times 100\%$. For the inclusions shown in Fig. 2, $r\left(1/K_{\text{sum}}^{2}\right)-1$ deviates from the $r\left(1/K_{\text{both}}^{2}\right)-1$ by more than 5% for $rD_{b}-1>0.34$ when using the first Born approximation, while the Monte Carlo and Rytov approximation results remain within 5% for all tested values of $rD_{b}-1$. Thus, we see that with spatial linearity taken into account, the Rytov approximation is in agreement with the Monte Carlo simulations and exhibits better linearity than the first Born approximation.

**Fig. 3.**
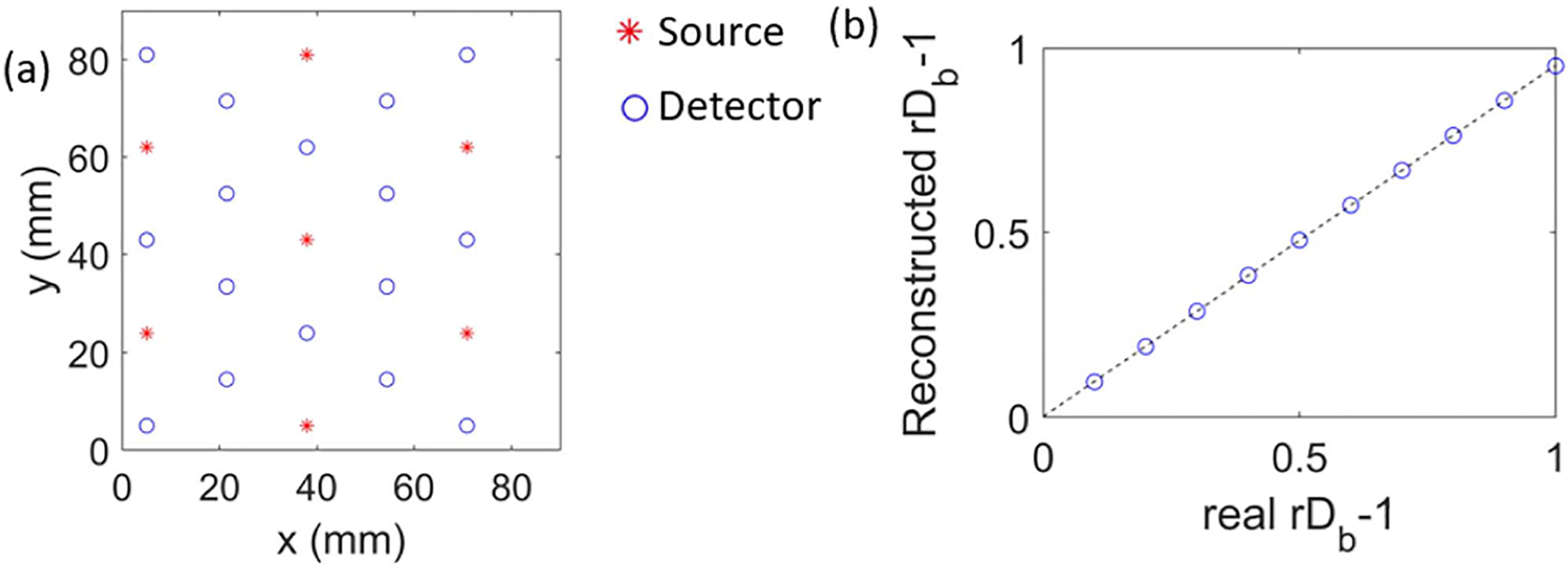
Validation of the linearity in the reconstructed versus true $rD_{b}$ (a) The hexagonal source-detector array used in the simulation. The nearest neighbor is 19 mm and the second nearest is 33mm. (b) The reconstructed versus real $rD_{b}-1$ within the volume. The inclusion is 40 × 40 × 40 mm^3^ at x=40mm, centered at y=40mm, and 10 mm beneath the surface of the sample. Reconstructed values are the average over the inclusion region. Other parameters are the same as those used in Fig. 1.

One last linearity test is the linearity in the reconstructed versus the true CBF change, which has been shown in absorption-based techniques to exhibit nonlinear effects when the first Born approximation is used [39]. With the above validations of the linearity in $r\left(1/K^{2}\right)-1$ versus $rD_{b}-1$, as well as the spatial linearity that the total $r\left(1/K^{2}\right)-1$ can be represented as the sum of the $r\left(1/K^{2}\right)-1$ in individual regions, we can construct a linear model for the forward problem. In principle, one can use the Monte Carlo simulations to construct the sensitivity matrix, but this requires injecting a large number of photons (10^8^ in this manuscript) and recording the locations of the scattering events within the medium, which is computationally expensive. Thus, we will use the Rytov approximation. To illustrate the image reconstruction method numerically, we first set the high-density source-detector probe design with 7 sources and 16 detectors on a hexagonal pattern following the design we had before for functional near infrared spectroscopy (fNIRS) [40], as shown in Fig. 3(a). A total of 50 channels are formed, considering the closest (19 mm) and the second-nearest (33 mm) source-detector separation channels. The third-nearest channels (50 mm) typically yield camera readings (e.g. around 0.5 camera count for our system) lower than camera read noise (e.g. around 1 camera count for our system) in human measurements. Thus, these and all channels with greater source-detector separations are excluded. We use Monte Carlo simulations to compute the forward problem of calculating $r\left(1/K^{2}\right)$ for each source-detector pair, and the Rytov approximation to obtain the A matrix. $rD_{b}$ is then obtained using (14). The inclusion is a $40\times 40\times 40\;{mm}^{3}$ at =40mm, y=40mm, and 10mm beneath the surface of the sample. We see that with the Rytov approximation, the reconstructed $rD_{b}-1$ behaves linearly for $rD_{b}-1$ changes from 0 to 1.

### B. Experimental Results

**Fig. 4.**
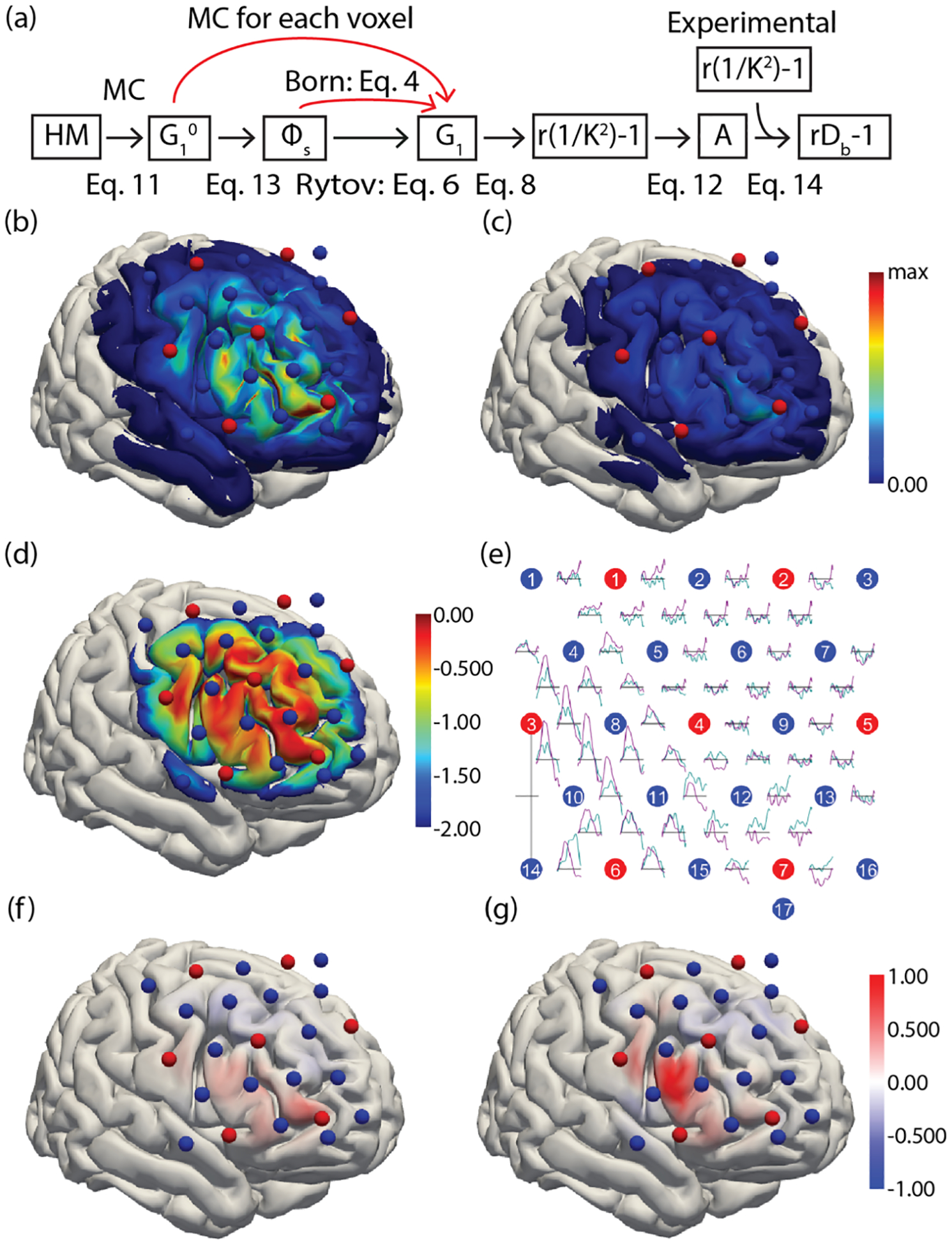
Image reconstruction of blood flow changes in the human brain from SCOS data in one subject using the Rytov approximation. (a) Schematic illustrating the steps involved in image reconstruction. Black arrows indicate steps used in this work; red arrows mark where the first Born approximation or Monte Carlo could have been used instead. HM, head model; MC, Monte Carlo; $G_{1}^{0}$, baseline field autocorrelation from MC; $\Phi_{s}$, perturbation term from (13); $G_{1}$, field autocorrelation after perturbation; $r\left(1/K^{2}\right)-1$, simulated change in $r\left(1/K^{2}\left(r_{s},r_{d};r^{\prime }\right)-1\right.$ from integrating the perturbed field autocorrelation; A, the sensitivity matrix $A\left(r_{s},r_{d};r^{\prime }\right)$ from (12); Experimental $r\left(1/K^{2}\right)-1$, the measured $r\left(1/K^{2}\left(r_{s},r_{d}\right)-1\right.$ from subject data; $rD_{b}-1$, the reconstructed relative change in blood flow at each voxel $r^{\prime }$. (b)-(c) Sum of $G_{1}^{0}\left(r_{s},r^{\prime },\tau \right)$ for all sources at each voxel, projected onto the brain surface for visualization. The result is shown with a decorrelation time τ of (b) 0.1μs and (c) 6μs. The color bar shows the strength of the correlation at each brain location. Blue spheres indicate probe detector optode locations and red spheres indicate probe source optode locations. (d) Visualization of the sensitivity matrix generated using the Rytov approximation. Plotted is the log base 10 of the sum of the sensitivity across all source-detector channels. (e) Channel space $r\left(1/K^{2}\right)-1$ map, showing data used for image reconstruction. Trial averages for congruent and incongruent word color stroop (WCS) task are plotted in cyan and magenta respectively. (f) WCS induced normalized $rD_{b}-1(t>10\;\text{s}\;\&\;t<15\;\text{s})$ reconstructed image for the congruent task. (g) WCS induced normalized $rD_{b}-1(t>10\;\text{s}\;\&\;t<15\;\text{s})$ reconstructed image for the incongruent task. Here the WCS task is between t=0 and =18s.

After validating the linearity of the SCOS signal $r\left(1/K^{2}\right)-1$ with the blood flow changes $rD_{b}-1$ theoretically, we perform image reconstruction of $rD_{b}-1$ using experimentally measured SCOS data. We show the reconstructed $rD_{b}-1$ duri`ng a WCS task in Fig. 4. The image reconstruction data analysis pipeline is illustrated in a schematic in Fig. 4(a). The black arrows indicate the steps used in this work, and the red arrows indicate where the first Born approximation or Monte Carlo could have been used instead for calculating the perturbed field autocorrelation. The first Born approximation was not used since we have demonstrated better linearity using the Rytov approximation and Monte Carlo simulations. The full Monte Carlo simulations were not used to generate the sensitivity matrix because it would be too slow. Using the Rytov or the first Born approximations, calculating the sensitivity matrix took 6.61 × 10^3^ seconds (~ 2 hours). Using Monte Carlo simulation to generate a sensitivity matrix would require simulating the effect of a perturbation at every voxel in the head model. The head model used in this work contains 7.11 × 10^6^ voxels. The time to run a single simulation instance is 65.0s, so it would take 6.50 × 10^1^ * 7.11 × 10^6^ = 4.62 × 10^8^ seconds (~ 14.6 years) to generate the sensitivity matrix using the Monte Carlo method. This further needs to be repeated once for each decorrelation time (129 in this work) when using (11) to calculate $G_{1}\left(r_{s},r^{\prime },\tau \right)$. Therefore, instead of simulating brain activations in every voxel, we only computed the baseline field autocorrelation, $G_{1}^{0}\left(r_{s},r^{\prime },\tau \right)$, using the Monte Carlo simulation, and calculated the brain activation using the Rytov approximation described in (5)–(6). The simulation results of the sum of $G_{1}^{0}\left(r_{s},r^{\prime },\tau \right)$ for all sources $r_{s}$ at two τ values are shown in Fig. 4(b), (c). A sensitivity matrix is generated based on (12), resulting in the visualization in Fig. 4(d). Here we used $\Delta D_{b}\left(r^{\prime }\right)=1\times 10^{-7}$ in the voxels to calculate the sensitivity matrix; the choice of this number does not affect the result for a linear system. We used $r\left(1/K^{2}\right)-1$ measured experimentally from one subject to reconstruct the blood flow changes. The results of $r\left(1/K^{2}\right)-1$ changes during brain activation measured in all the source-detector channels are shown in Fig. 4(e). Resulting plots showing normalized $rD_{b}-1$ are calculated from (14). Results for $rD_{b}-1$ dynamics are shown for both the congruent, Fig. 4(f), and the incongruent, Fig. 4(g), WCS task. As expected, there is a greater relative activation observed in the harder incongruent task compared to the easier congruent task. Although no quantitative conclusions can be drawn from the measurement of a single subject, this result demonstrates the application of the linear image reconstruction method described in this manuscript to experimental SCOS/SCOT data.

## IV. Conclusion

In summary, we have validated the image reconstruction methods using the Rytov approximation as compared with the Monte Carlo simulations, and show that it exhibits better linearity and accuracy than the first Born approximation used in the prior studies. We have demonstrated linearity (1) in $r\left(1/K^{2}\right)$ versus $rD_{b}$, (2) spatially, and (3) in the reconstructed versus true $rD_{b}$ variations. Given this validation, we showcase reconstruction of multi-channel SCOS data for a subject using the Rytov approximation. In addition to image reconstruction, the results also indicate that many linear methods developed for functional near-infrared spectroscopy (fNIRS), including short-separation regression using the general linear model [41], can potentially be applied to SCOS/SCOT data analysis, which can be compared with non-linear models [42]. Theoretically, the linearity of the Rytov approximation will also break down. We found that at around $rD_{b}=10$, the reconstructed $rD_{b}$ starts to deviate from the linear behavior, but physiologically we will not experience such a large $rD_{b}$ change. Here we have ignored absorption changes in the model since the main focus is to test the linearity in the SCOT signal versus CBF changes, therefore we utilized a simplified tissue model of a uniform semi-infinite scattering medium with known analytical solutions for the linearity tests in Figs. 1–3. We expect that simultaneous reconstruction of the absorption and blood flow changes could further improve the accuracy which will be explored in the future for human brain function measurements.

## Figures and Tables

**TABLE I:** Table of the Optical and Dynamic Properties for the Anatomical Model Representing the Skin, Skull, Dura Matter (DM), Cerebrospinal Fluid (CSF), Gray Matter (GM), and White Matter (WM)

	$\mu_{a}\;\left(1/mm\right)$	$\mu_{s}^{\prime }\;\left(1/mm\right)$	${\alpha D}_{b}\;\left({mm}^{2}/s\right)$
Skin	0.0191	0.65934	1 × 10^−6^
Skull	0.0136	0.85914	1 × 10^−6^
DM	0.0191	0.65934	1 × 10^−6^
CSF	0.0026	0.00999	1 × 10^−6^
GM	0.0186	1.0989	6 × 10^−6^
WM	0.0186	0.85914	6 × 10^−6^
